# ATGL activity regulates GLUT1-mediated glucose uptake and lactate production *via* TXNIP stability in adipocytes

**DOI:** 10.1016/j.jbc.2021.100332

**Published:** 2021-01-27

**Authors:** Muheeb Beg, Wei Zhang, Andrew C. McCourt, Sven Enerbäck

**Affiliations:** Department of Medical Biochemistry and Cell Biology, Institute of Biomedicine, Sahlgrenska Academy, University of Gothenburg, Gothenburg, Sweden

**Keywords:** glucose transport, cAMP, adipose triglyceride lipase (ATGL), PKA, adipocyte, triacylglycerol, thioredoxin-interacting protein (TXNIP), hormone-sensitive lipase (HSL), 8-Br-cAMP, 8-bromine-cAMP, AKT, protein kinase B, AMPK, AMP-activated protein kinase, ATGL, adipose triglyceride lipase, DOG, 2-deoxy-d-glucose, FFA, free fatty acid, GLUT, glucose transporter, GSK3, glycogen synthase kinase 3, HA, human influenza hemagglutinin, HSL, hormone-sensitive lipase, MGL, monoacylglycerol lipase, OE, overexpression, TAG, triacylglycerol, TXNIP, thioredoxin-interacting protein

## Abstract

Traditionally, lipolysis has been regarded as an enzymatic activity that liberates fatty acids as metabolic fuel. However, recent work has shown that novel substrates, including a variety of lipid compounds such as fatty acids and their derivatives, release lipolysis products that act as signaling molecules and transcriptional modulators. While these studies have expanded the role of lipolysis, the mechanisms underpinning lipolysis signaling are not fully defined. Here, we uncover a new mechanism regulating glucose uptake, whereby activation of lipolysis, in response to elevated cAMP, leads to the stimulation of thioredoxin-interacting protein (TXNIP) degradation. This, in turn, selectively induces glucose transporter 1 surface localization and glucose uptake in 3T3-L1 adipocytes and increases lactate production. Interestingly, cAMP-induced glucose uptake *via* degradation of TXNIP is largely dependent upon adipose triglyceride lipase (ATGL) and not hormone-sensitive lipase or monoacylglycerol lipase. Pharmacological inhibition or knockdown of ATGL alone prevents cAMP-dependent TXNIP degradation and thus significantly decreases glucose uptake and lactate secretion. Conversely, overexpression of ATGL amplifies the cAMP response, yielding increased glucose uptake and lactate production. Similarly, knockdown of TXNIP elicits enhanced basal glucose uptake and lactate secretion, and increased cAMP further amplifies this phenotype. Overexpression of TXNIP reduces basal and cAMP-stimulated glucose uptake and lactate secretion. As a proof of concept, we replicated these findings in human primary adipocytes and observed TXNIP degradation and increased glucose uptake and lactate secretion upon elevated cAMP signaling. Taken together, our results suggest a crosstalk between ATGL-mediated lipolysis and glucose uptake.

Adipose tissue metabolism plays a critical role in maintaining healthy homeostasis for glucose and lipid metabolism (1). Insulin mediated triacylglycerol (TAG) synthesis, and its dissipation through lipolysis is an important physiological process, which is tightly controlled and under metabolic regulation (2). When in positive energy balance, during the postprandial period, insulin rapidly stimulates glucose transporter 4 (GLUT4) surface translocation, which mediates glucose uptake. This is an acute insulin-stimulated process that is followed by TAG formation (3). In a similar way, the counter-regulatory process during extended periods of starvation, through the action of the lipases, adipose triglyceride lipase (ATGL), hormone-sensitive lipase (HSL), and monoacylglycerol lipase (MGL), lipolysis is activated by elevated levels of cAMP, which ultimately leads to the release of free fatty acid (FFA) (4, 5). Mice with a targeted deletion of the various lipases display a vast range of phenotypes involving release of FFA, which are dependent upon tissue specificity and diet composition (6, 7, 8). Interesting studies in this area include the roles of diacylglycerol and ceramide, reported to negatively regulate glucose uptake and lead to insulin resistance (9, 10). Similarly, lipid derivatives, such as arachidonic acid and the recently discovered hydroxylated fatty acid, positively regulate glucose uptake (11, 12). In conclusion, lipolytic activity is not only a mechanism to release FFA but also it is increasingly clear that these activities play an important role in regulating other modalities of metabolism (4).

Glucose uptake is fundamental for cell metabolism and is mediated through a set of GLUTs and their associated machinery. Specialized cells, such as adipocytes, express GLUT4, which is largely dependent on insulin signaling for activation and surface translocation (13, 14). Another transporter, GLUT1, is more ubiquitously expressed and is also important for adipocyte glucose uptake. Unlike GLUT4, the machinery, signaling, and metabolic regulation in relation to GLUT1 are less studied. Some of the central molecules for the glucose uptake process, such as AS160 phosphorylation and thioredoxin-interacting protein (TXNIP) expression levels, which are directly linked to glucose uptake, are regulated by both anabolic and catabolic signals and affect both GLUT1 and GLUT4 transporter surface translocation (15, 16). The adipocyte expresses both GLUT1 and GLUT4, together with several lipases, and can hence be considered a rheostat for glucose and FFA metabolism that responds to a variety of both anabolic and catabolic signals.

Here, we show that elevated levels of cAMP rapidly degrade TXNIP in a dose-dependent manner and thereby release GLUT1 for surface localization. GLUT1 surface localization stimulates glucose uptake, which to a large extent is further metabolized to lactate. We uncover a mechanism by which ATGL activity regulates cAMP-dependent TXNIP degradation that in turn regulates GLUT1-, but not GLUT4-, mediated glucose uptake in adipocytes.

## Results

### cAMP-dependent TXNIP degradation induces glucose uptake in adipocytes and functions independently of AKT and AMPK pathways but is dependent on PKA

**Figure 1:**
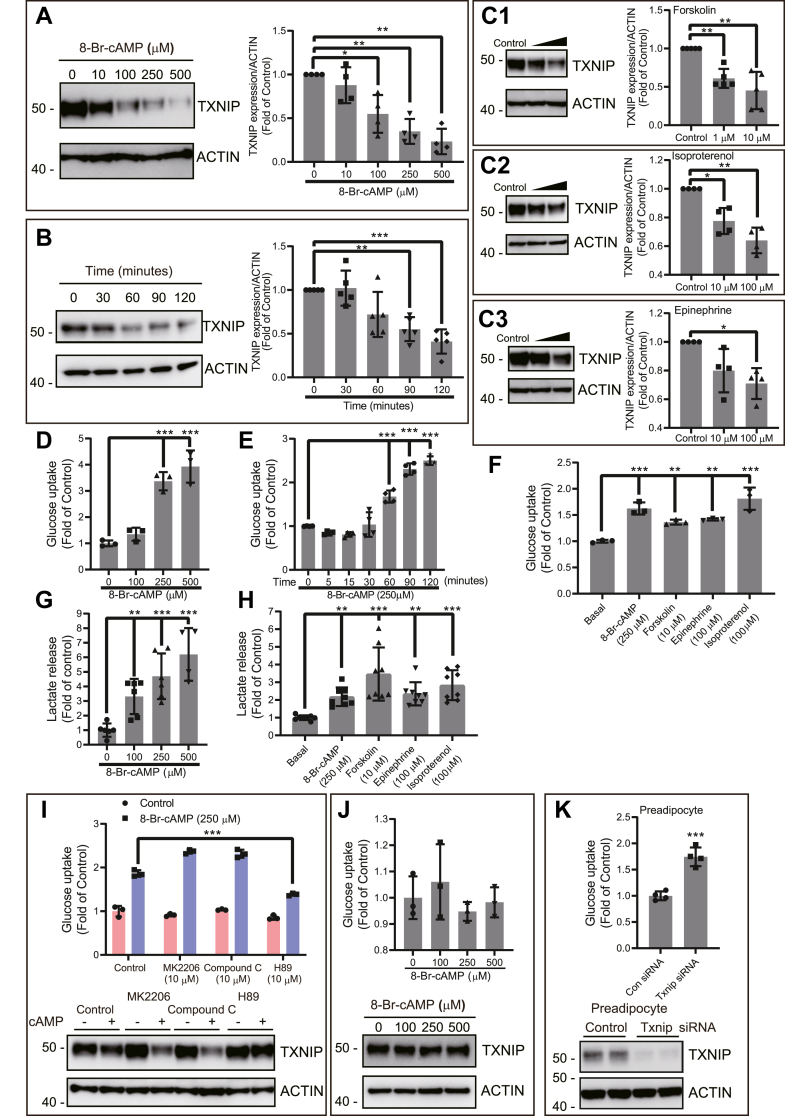
**Elevated levels of cAMP rapidly degrade TXNIP and cause glucose uptake and lactate secretion.***A*–*C*, differentiated adipocyte cells were treated with 8-Br-cAMP in (*A*) dose-dependent manner for 2 h or (*B*) treated for various time points (30–120 min) or treated with (*C1*–*C3*) cAMP agonists, *C1* with forskolin, *C2* with isoproterenol, and *C3* with epinephrine, for 2 h. Cell lysates were collected and immunoblotted for TXNIP expression levels. Representative blots are shown with densitometric analysis drawn from three to five biologically independent experiments. Beta actin was used as a housekeeping protein, and values were subtracted for analysis. *D*–*F*, in a similar experimental setting, ^3^H-2-DOG glucose uptake was determined after (*D*) dose-dependent (N = 5) or (*E*) time-dependent (N = 3) treatment of 8-Br-cAMP and (*F*) cAMP-stimulating agents (N = 4). All glucose uptake values were normalized to protein measurements. *G* and *H*, differentiated adipocyte cells after 2 h treatment with (*G*) various doses of 8-Br-cAMP (N = 6) or (*H*) cAMP-stimulating agents (N = 4), small aliquots of supernatant medium were taken and measured for secreted lactate content. Lactate secretion values were normalized to protein measurement. *I*, effect of MK2206, compound C, and H89 on cAMP-stimulated ^3^H-2-DOG glucose uptake was determined by pretreating cells with these inhibitors for 20 min followed by 2 h of cAMP treatment (N = 3). *Lower panel*, in a similar setting, cell lysates were collected and immunoblotted to assess TXNIP expression changes (N = 3). *J*, to measure the effect of cAMP-mediated glucose uptake in preadipocytes, 3T3 preadipocytes were treated with various doses of cAMP, followed by ^3^H-2-DOG glucose uptake measurement (N = 2). In a similar setting, cell lysates were collected to assess TXNIP expression changes. *K*, preadipocytes were assessed for glucose uptake following TXNIP KD. *Lower panel* shows the TXNIP KD level in the preadipocyte cell lysate (N = 3). For glucose uptake, results shown are normalized mean ± SD of representative experiment. For lactate measurement, results are normalized mean ± SD of three to six biologically independent experiments. In all experiments, N represents biologically independent repeats. *A*–*C*, one-sample *t* test against one as a theoretical mean was used for statistical significance. Ordinary one-way ANOVA statistics was used for *D*–*H* and *J*. Two-way ANOVA statistics was used for *I*. Holm–Sidak multiple comparison test was used to compare the selected pairs of means. Significance was marked as the pairwise comparisons on the figure. ∗∗∗*p* < 0.001, ∗∗*p* < 0.01, and ∗*p* < 0.05. 8-Br-cAMP, 8-bromine-cAMP; ^3^H-2-DOG, ^3^H-2-deoxy-d-glucose; TXNIP, thioredoxin-interacting protein.

Besides providing glycerol and fatty acids, lipolysis also generates several lipid derivatives and substrate molecules that regulate metabolism through multiple mechanisms, including gene transcription and post-translational modifications (4, 5). A good example hereof are studies in which diacylglycerol, ceramide, and saturated fatty acids, all linked to lipolysis, have been shown to regulate metabolism (4, 9, 10, 17). We found that TXNIP, a gatekeeper protein for GLUTs, is rapidly degraded following 8-bromine-cAMP (8-Br-cAMP) (hereafter also termed as cAMP) treatment in adipocytes. We performed concentration- and time-dependent experiments with 8-Br-cAMP to determine the effective dose and period. We observed that 100 to 250 μM cAMP concentration for 2 h significantly reduced TXNIP at the protein level (Fig. 1*A*). We also exposed 3T3-L1 adipocytes to 8-Br-cAMP in a time-dependent manner up to 2 h and found that degradation of TXNIP initiated from 60 min onward (Fig. 1*B*). To determine whether other standard stimuli that increase the level of cAMP also cause degradation of TXNIP, we used multiple standard stimuli, such as forskolin, isoproterenol, and epinephrine. We found that, in parallel with 8-Br-cAMP, forskolin, isoproterenol, and epinephrine also significantly degrade TXNIP levels (Fig. 1, *C1*–*C3*). Interestingly, cAMP-stimulated TXNIP protein expression changes are not reflected in mRNA levels, suggesting that cAMP-mediated TXNIP degradation is a post-translational event (Fig. S1). TXNIP, being a regulator of glucose uptake, retains and internalizes GLUTs, thus preventing their surface localization. TXNIP degradation has been linked to the uptake of glucose (16). In a next experiment, we measured ^3^H-2-deoxy-d-glucose (DOG) uptake. We found that 8-Br-cAMP, as expected, increased glucose uptake both in a concentration- and time-dependent manner, which is paralleled with TXNIP degradation (Fig. 1, *D* and *E*). Similar to that observed with 8-Br-cAMP, administration of other cAMP agonists, such as forskolin, isoproterenol, and epinephrine, also showed increased glucose uptake in 3T3-L1 adipocytes (Fig. 1*F*).

We took an aliquot of supernatant after 2 h of cAMP incubation and measured the lactate level. We found that, similar to that with glucose uptake, lactate production is concomitantly increased with increasing concentrations of cAMP, and this was also found for other cAMP agonists (Fig. 1, *G* and *H*). Both protein kinase B (AKT) and AMP-activated protein kinase (AMPK) have previously been shown to regulate glucose uptake *via* TXNIP degradation (15, 16). We therefore tested whether AKT and AMPK inhibition could control cAMP-mediated glucose uptake. Coincubation with the inhibitors, MK2206 (AKT) or compound C (AMPK), did not reduce either the cAMP-mediated glucose uptake in 3T3-L1 adipocytes or TXNIP degradation (Fig. 1*I*, *lower panel*). These data suggest that cAMP acts *via* another pathway. Furthermore, we also tested insulin signaling activation, which was found to be unchanged (Fig. S2), and further agrees with our AKT inhibition data (Fig 1*I*). We observed that the PKA inhibitor, H89, significantly reduces cAMP-mediated glucose uptake in 3T3-L1 adipocytes and TXNIP degradation (Fig. 1*I*, *lower panel*). Interestingly, these effects were present in neither 3T3-L1 preadipocytes (Fig. 1*J*, *lower panel*) nor C2C12 myoblasts, two cell types lacking significant intracellular triglyceride stores and associated signaling pathways (Fig. S3). However, knockdown of TXNIP in preadipocytes using an siRNA approach yielded a similar upregulation of glucose uptake as observed in adipocytes in response to cAMP-mediated TXNIP degradation (Fig. 1*K*, *lower panel*). These data support the role of TXNIP as a potent regulator of glucose uptake. Since cells not responding to cAMP with increased glucose uptake (preadipocytes and C2C12 cells) lack lipid droplets and associated pathways, which appear pivotal for TXNIP degradation, it is possible that they therefore could not upregulate glucose uptake in response to cAMP, despite the presence of the GLUT1 transporter (Fig. S4). Moreover, ectopic ATGL expression in preadipocytes was not sufficient to induce cAMP responsiveness as a signal for TXNIP degradation and glucose uptake (Fig. S5, *A* and *B*).

Elevated cAMP levels in adipocytes also leads to phosphorylation of glycogen synthase kinase 3 (GSK3) to initiate glycogenolysis. To investigate whether GSK3 phosphorylation contributes to the lactate production observed (Fig. 1*G*) by providing internal glucose substrate for lactate secretion, we used the GSK3-specific inhibitor, SB216763. We found that GSK inhibition does not cause any broad alteration in the cAMP response, which makes any direct involvement of GSK3 kinase unlikely (Fig. S6, *A* and *B*). Finally, to rule out any off-target effects of 8-Br-cAMP because of the chemical 8-Br moiety, or any similar overlapping pathway activated by cGMP, we used the similar analog, 8-Br-cGMP. We observed that 8-Br-cGMP did not show any alteration at the dose that is effective for 8-Br-cAMP (Fig. S7).

### The ATGL inhibitor atglistatin inhibits cAMP-mediated glucose uptake and TXNIP degradation

**Figure 2:**
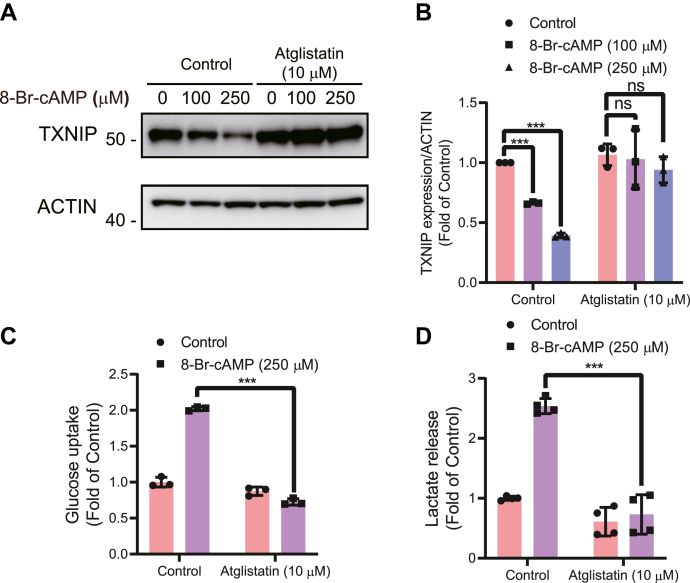
**Degradation of TXNIP and ensuing glucose uptake and lactate secretion are dependent upon cAMP-mediated lipolysis.***A* and *B*, differentiated adipocytes were pretreated with vehicle or atglistatin for 20 min followed by coincubation of 8-Br-cAMP for the next 2 h. After completion, cell lysates were collected and immunoblotted for TXNIP expression levels. *B*, densitometric analysis was performed using ImageJ software. Four biologically independent experiments were used to derive the densitometric values. Beta actin values were used as a normalization control. *C*, ^3^H-2-DOG glucose uptake (N = 5) and (*D*) lactate secretion (N = 5) determination after pretreatment of vehicle or atglistatin for 20 min following the presence or the absence of 2 h of cAMP treatment. Protein values were used for normalization and presented as CPM/microgram of protein. Glucose uptake and lactate secretion values were normalized to total protein content. For glucose uptake, results shown are normalized mean ± SD of representative experiment. For lactate measurement, results are normalized mean ± SD of three to six biologically independent experiments. In all experiments, N represents biologically independent repeats. *B*, one-sample *t* test against one as theoretical mean was used for non-atglistatin treatment comparisons, and unpaired *t* test was used for atglistatin treatment comparisons. Two-way ANOVA statistics was used for *C* and *D*. Holm–Sidak multiple comparison test was used to compare the selected pairs of means. Significance was marked as the pairwise comparisons on the figure. ∗∗∗*p* < 0.001, ∗∗*p* < 0.01, and ∗*p* < 0.05. 8-Br-cAMP, 8-bromine-cAMP; ^3^H-2-DOG, ^3^H-2-deoxy-d-glucose; TXNIP, thioredoxin-interacting protein.

We next used atglistatin, a specific inhibitor of ATGL, which is the first lipase activated in response to elevated cAMP—primary signal for lipolysis (18, 19). Interestingly, we found that atglistatin seems to block the cAMP-mediated TXNIP degradation as shown by immunoblot and densitometry analysis (Fig. 2, *A* and *B*). As hypothesized, and linked to TXNIP degradation, we found that atglistatin also completely abolished the cAMP stimulatory effect on glucose uptake and lactate production (Fig. 2, *C* and *D*). We further investigated the potency of atglistatin in inhibiting lipolysis by measuring the lipolytic end product, glycerol. We measured atglistatin-mediated ATGL suppression by both varying cAMP concentration and using several doses of atglistatin (Fig. S8, *A* and *B*). We observed that atglistatin robustly inhibited the cAMP-stimulated glycerol secretion without affecting HSL phosphorylation in the same setting, suggesting that atglistatin specifically blocks ATGL activity as previously reported (18, 20) (Fig. S8*C*).

In conclusion, these results indicate that ATGL activity is necessary for cAMP-induced and TXNIP degradation–mediated glucose uptake in 3T3-L1 adipocytes. It further suggests a link between glucose and lipid metabolism regulated by ATGL activity.

### ATGL, but not HSL or MGL, is largely responsible for cAMP-mediated and TXNIP degradation–dependent glucose uptake and lactate secretion in 3T3-L1 adipocytes

**Figure 3:**
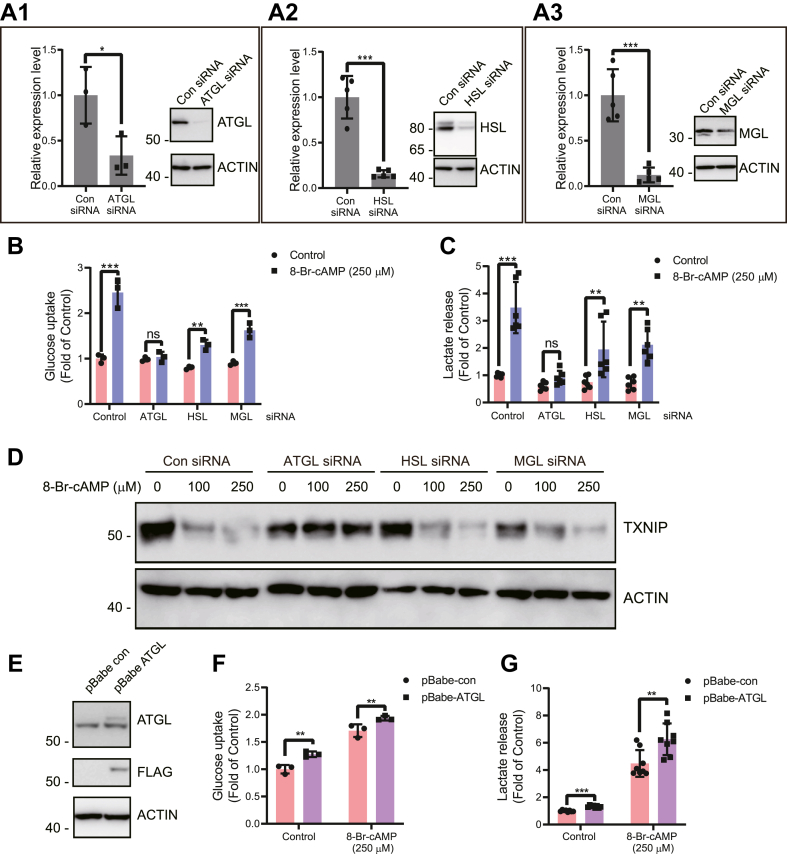
**ATGL-mediated lipolysis determines the cAMP-mediated increase in glucose uptake, lactate secretion, and TXNIP degradation.***A1*–*A3*, differentiated adipocyte cells were knocked down for (*A1*) ATGL, (*A2*) HSL, and (*A3*) MGL using an siRNA approach, and knockdown was confirmed by quantitative RT-PCR (on the *left side*) for all lipases (N = 3). Immunoblot level on the *right side* was shown. *B* and *C*, differentiated cells were knocked down for ATGL, HSL, and MGL using specific siRNAs, and knockdown cells were then treated with cAMP for 2 h and (*B*) ^3^H-2-DOG glucose uptake (N = 4) and (*C*) lactate secretion (N = 4) were determined. *D*, similar experimental settings were used to assess the TXNIP expression changes (N = 4). *E*, 3T3 adipocytes overexpressing ATGL were immunoblotted to confirm overexpression of ATGL. *F* and *G*, differentiated adipocytes overexpressing ATGL or empty vector were assessed for (*F*) glucose uptake (N = 4) and (*G*) lactate secretion (N = 4) in response to cAMP treatment. Glucose uptake and lactate secretion values were normalized to total protein content. For glucose uptake, results shown are normalized mean ± SD of representative experiment. For lactate measurement, results are normalized mean ± SD of three to six biologically independent experiments. In all experiments, N represents biologically independent repeats. Unpaired two-sided Student's *t* test was used for *A* and *G*. Two-way ANOVA was used for *B*, *C*, and *F*. Holm–Sidak multiple comparison test was used to compare the selected pairs of means. Significance was marked as the pairwise comparisons on the figure. ∗∗∗*p* < 0.001, ∗∗*p* < 0.01, and ∗*p* < 0.05. ATGL, adipose triglyceride lipase; ^3^H-2-DOG, ^3^H-2-deoxy-d-glucose; HSL, hormone-sensitive lipase; MGL, monoacylglycerol lipase; TXNIP, thioredoxin-interacting protein.

Cellular lipolysis is essentially carried out by three lipases: ATGL, HSL, and MGL, which work in concert to break down TAG to fatty acids and glycerol. Our data suggest that lipolysis leads to degradation of TXNIP, and linked to this, increased glucose uptake and lactate secretion. To study the relative contribution of these three lipases to the observed phenotype of cAMP-mediated glucose uptake, lactate secretion, and TXNIP degradation, we knocked down ATGL, HSL, or MGL in 3T3-L1 adipocytes (Fig. 3, *A1*–*A3*). In agreement with our previous results, we found that cAMP-mediated glucose uptake is largely regulated through ATGL activity as knockdown of ATGL almost completely abrogated the cAMP-mediated effects. This is very similar to the atglistatin effect, which is a specific inhibitor of ATGL (Fig. 2, *C* and *D*). Unlike ATGL, HSL and MGL knockdown still maintain a significant response to cAMP in terms of glucose uptake and lactate production (Fig. 3, *B* and *C*). In line with these findings, we could demonstrate that knockdown of only ATGL led to significant inhibition of TXNIP degradation (Fig. 3*D*). We further validated our results with stable knockdown for ATGL though a specific shRNA approach. We observed that, similar to atglistatin and siRNA knockdown, shRNA-mediated knockdown of ATGL completely abrogates the cAMP-mediated glucose uptake and inhibits TXNIP degradation (Fig. S9, *A* and *B*).

Overexpression (OE) of ATGL in 3T3-L1 adipocytes elicits a significant increase in glucose uptake and lactate secretion (Fig. 3, *E*–*G*). This further supports the notion that ATGL-mediated lipolysis is necessary for cAMP-induced glucose uptake and lactate secretion.

### cAMP-mediated glucose uptake is mediated through GLUT1 but not GLUT4 surface localization

**Figure 4:**
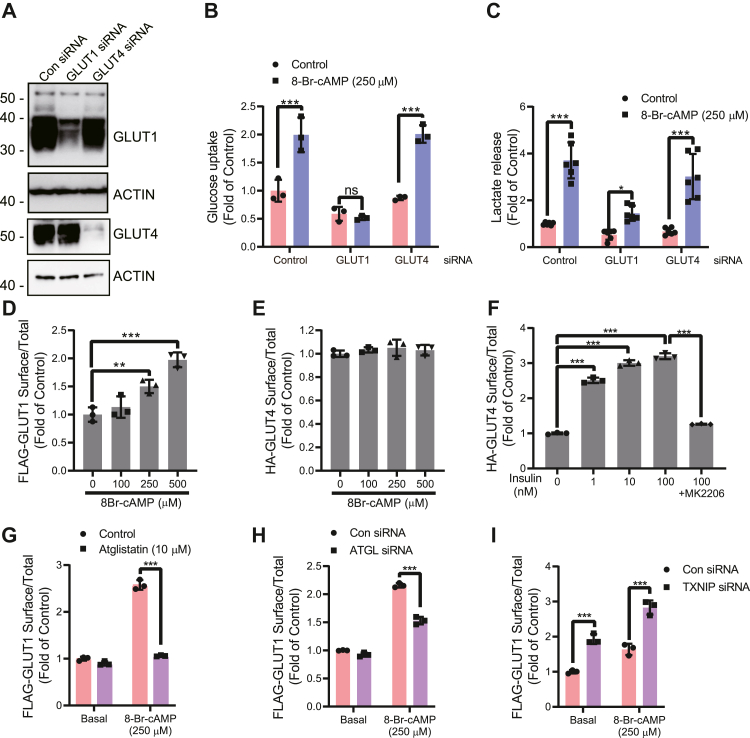
**cAMP-mediated lipolysis causes surface localization of GLUT1 but not GLUT4.***A*, differentiated adipocytes were subjected to GLUT1 and GLUT4 siRNA, and immunoblot was used to confirm knockdown (N = 3). *B* and *C*, differentiated adipocyte cells were knocked down for GLUT1 and GLUT4 and (*B*) ^3^H-2-DOG glucose uptake (N = 4) and (*C*) lactate secretion (N = 3) determined after the presence or the absence of cAMP treatment for 2 h. *D*–*G*, FLAG-GLUT1 was cloned into pBabe retroviral vector to generate stable expression of tagged GLUT1 into 3T3-L1 preadipocyte. Stably tagged FLAG-GLUT1–expressing cells were differentiated and treated with (*D*) various concentrations of cAMP (N = 3) for 2 h followed by cell fixation. GLUT1 surface/total ratio was determined as described in Experimental procedures section and presented as fold stimulation. *E* and *F*, HA-GLUT4 was cloned into pBabe vector to stably express tagged GLUT4. Tagged GLUT4–expressing cells were differentiated and GLUT4 surface/total ratios were determined in response to (*E*) various increasing doses of cAMP for 2 h (N = 2) and (*F*) increasing dose of insulin stimulation for the last 20 min during the 2 h of incubation. Cells were treated with MK2206 (10 μM) to show that AKT inhibition blocks insulin-stimulated GLUT4 translocation (N = 2). *G*–*I*, to assess the cAMP-mediated GLUT1 surface localization upon pharmacological inhibition or knockdown of ATGL or TXNIP knockdown, adipocytes pretreated with (*G*) atglistatin (N = 5) and cells that were knocked down for (*H*) ATGL (N = 3) and (*I*) TXNIP protein (N = 3) were treated with cAMP for 2 h followed by cell fixation and staining. For glucose uptake and GLUT1 and GLUT4 surface localization experiments, results shown are normalized mean ± SD of representative experiment. For lactate measurements, results are normalized mean ± SD of three to six biologically independent experiments. In all experiments, N represents biologically independent repeats. Ordinary one-way ANOVA statistics was used for *D*–*F*. Two-way ANOVA statistics was used for *B*, *C*, *G*–*I*. Holm–Sidak multiple comparison test was used to compare the selected pairs of means. Significance was marked as the pairwise comparisons on the figure. ∗∗∗*p* < 0.001, ∗∗*p* < 0.01, and ∗*p* < 0.05. ATGL, adipose triglyceride lipase; ^3^H-2-DOG, ^3^H-2-deoxy-d-glucose; GLUT, glucose transporter; TXNIP, thioredoxin-interacting protein.

GLUTs are the facilitative transporters for glucose uptake. Adipocytes express both GLUT1 and GLUT4. GLUT4 is a well-studied insulin-regulated transporter, whereas GLUT1 regulation in adipocytes is still not fully understood (21, 22, 23). TXNIP serves as a gatekeeper for glucose uptake through internalizing both GLUT1 and GLUT4 (15, 16, 24). We used specific siRNAs to knockdown GLUT1 or GLUT4 to investigate the roles of GLUT1 and GLUT4 in mature 3T3-L1 adipocytes (Fig. 4*A*). We found that GLUT1 knockdown completely abolished cAMP-mediated glucose uptake, whereas GLUT4 knockdown had essentially no effect on cAMP-mediated glucose uptake in 3T3-L1 adipocytes as compared with a control siRNA (Fig. 4*B*). Similar trends were found for lactate release (Fig. 4*C*). This suggests that cAMP acts *via* ATGL and that this triggers a TXNIP degradation that specifically stimulates GLUT1 but not GLUT4 surface localization. We also adapted a complementary approach using stable knockdown of GLUT1 through shRNA in adipocytes and observed, as expected, a blunting of cAMP-mediated glucose uptake (Fig. S10). Thus, the cAMP-mediated and TXNIP degradation–linked increase in glucose uptake in 3T3-L1 adipocytes is mediated by GLUT1 and not GLUT4.

As surrogate markers for surface localization of GLUTs, we used GLUT1 and GLUT4 tagged with FLAG and human influenza hemagglutinin (HA), respectively. In 3T3-L1 adipocytes, transfected to stably express these constructs, we used an indirect ELISA method to quantitate the surface to total ratio of GLUT1 and GLUT4. Mimicking previous glucose uptake experiments (Fig. 1*D*), we observed a dose-dependent increase in 8-Br-cAMP–linked increase in surface localization of GLUT1 (Fig. 4*D*) but not of GLUT4 (Fig. 4*E*). HA–GLUT4 constructs remain unresponsive to cAMP stimulation, however, as expected, insulin rapidly caused GLUT4 translocation in a dose-dependent manner, which was abolished by the AKT inhibitor, MK2206 (Fig. 4*F*). Furthermore, we tested whether cAMP-mediated GLUT1 surface localization was affected by pharmacological inhibition or knockdown of ATGL. We also found that ATGL inhibition by atglistatin or ATGL knockdown significantly reduces cAMP-mediated GLUT1 surface localization (Fig. 4, *G* and *H*). Moreover, we found that TXNIP knockdown significantly upregulated GLUT1 surface localization and that was further amplified by 8-Br-cAMP (Fig. 4*I*). In conclusion, it appears that the increase in glucose uptake in 3T3-L1 adipocytes mediated by cAMP stimulation and linked to TXNIP degradation is mediated by GLUT1 but not GLUT4.

### TXNIP expression level is a major determinant for GLUT1-mediated glucose uptake in both the basal and cAMP-stimulated state

**Figure 5:**
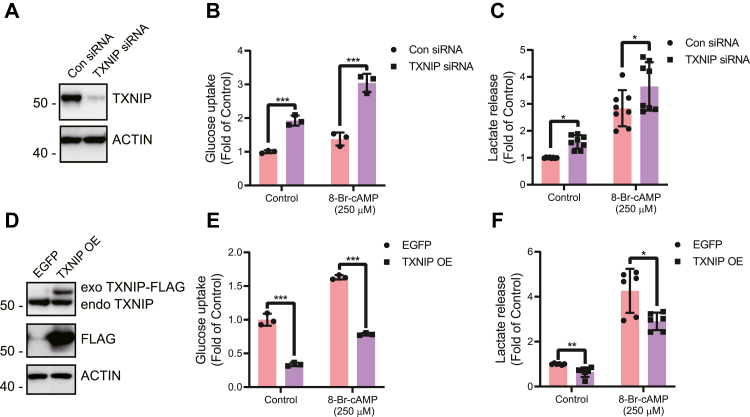
**TXNIP serves as a pivotal center for glucose uptake and lactate secretion.***A*–*C*, differentiated adipocytes were knocked down for TXNIP and confirmed with (*A*) immunoblotting. TXNIP knockdown adipocyte cells were assessed for cAMP-mediated (*B*) glucose uptake (N = 4) and (*C*) lactate secretion (N = 4). *D*–*F*, differentiated adipocytes stably expressing control vector enhanced GFP or overexpressing TXNIP were (*D*) immunoblotted for TXNIP. TXNIP-overexpressing cells were differentiated and assessed for cAMP-mediated (*E*) glucose uptake (N = 4) and (*F*) lactate secretion (N = 4). Glucose uptake and lactate secretion values were normalized to total protein content. For glucose uptake, results shown are normalized mean ± SD of representative experiment. For lactate measurements, results are normalized mean ± SD of three to six biologically independent experiments. In all experiments, N represents biologically independent repeats. Unpaired two-sided Student's *t* test was used for *F*. Two-way ANOVA statistics was used for *B*, *C*, and *E*. Holm–Sidak multiple comparison test was used to compare the selected pairs of means. Significance was marked as the pairwise comparisons on the figure. ∗∗∗*p* < 0.001, ∗∗*p* < 0.01, and ∗*p* < 0.05. TXNIP, thioredoxin-interacting protein.

**Figure 6:**
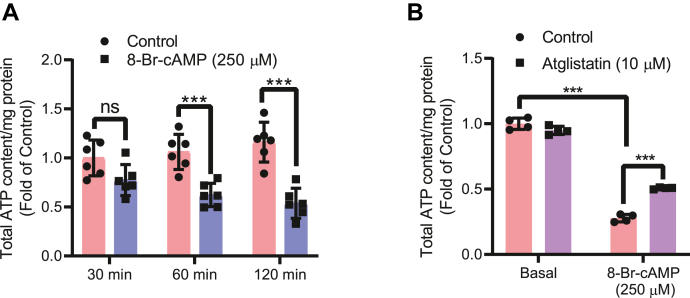
**cAMP-stimulated depletion of ATP might be responsible for enhancing glycolysis.***A* and *B*, differentiated adipocyte cells were treated with cAMP in (*A*) a time-dependent manner (N = 3) or (*B*) pretreated with atglistatin for 20 min (N = 3). Cell lysates were collected, and ATP measurements were performed as per manufacturer's protocol. All values were then subtracted with the corresponding protein well and presented as total ATP/milligram protein. For ATP measurements, results are normalized mean ± SD of three biologically independent experiments. Two-way ANOVA statistics was used for *A* and *B*. Holm–Sidak multiple comparison test was used to compare the selected pairs of means. Significance was marked as the pairwise comparisons on the figure. ∗∗∗*p* < 0.001, ∗∗*p* < 0.01, and ∗*p* < 0.05.

We used siRNA to knockdown TXNIP expression in 3T3-L1 adipocytes (Fig. 5*A*) and observed that TXNIP knockdown significantly upregulated glucose uptake in the basal state and also significantly amplified the cAMP response (Fig. 5*B*). There was also a modest effect on lactate secretion (Fig. 5*C*). In a next step, we used retrovirus to stably overexpress mouse TXNIP with a FLAG tag (Fig. 5*D*). TXNIP OE reduced glucose uptake in both the basal and cAMP-stimulated state (Fig. 5*E*), which was in stark contrast to the TXNIP knockdown phenotype (Fig. 5*B*). Similarly, lactate secretion was significantly downregulated (Fig. 5*F*). Enhanced lactate secretion, a sign of stimulated glycolysis, might be a way to rapidly replete the total cellular ATP pool. Thus, we observed that cAMP, in a time-dependent manner, reduced the total ATP pool (Fig. 6*A*), which is in concurrence with TXNIP degradation (Fig. 1*B*), glucose uptake (Fig. 1*E*), and lactate secretion (Fig. 1*G*) data. Moreover, coincubation with an ATGL inhibitor restricted the decrease in the ATP pool (Fig. 6*B*). Overall, these evidences suggest that adipocytes, in response to acute elevated cAMP levels, upregulate glycolysis, which in turn acts to replenish the ATP pool.

### Primary human adipocytes show similar phenotypic changes upon elevated cAMP signaling

**Figure 7:**
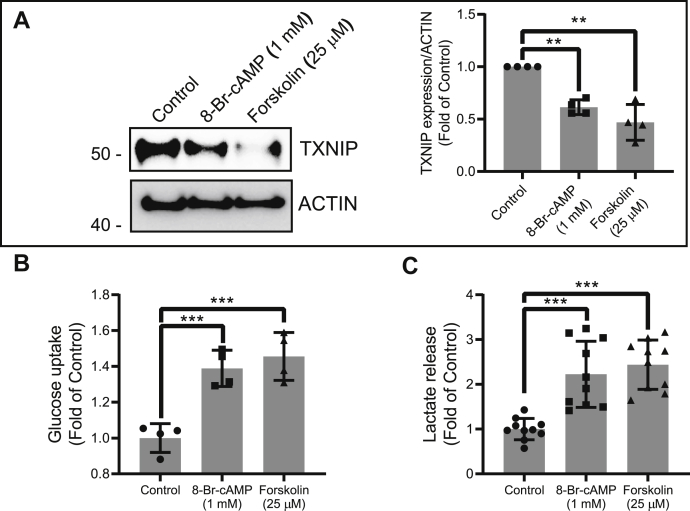
**Differentiated primary human adipocytes replicate the same phenotype upon elevated cAMP signaling.***A*, differentiated primary human adipocytes were treated with cAMP and forskolin for 2 h, and cell lysates were collected and run for immunoblot analysis of TXNIP. *Right panel*, quantification was performed on four independent experiments using ImageJ software. Differentiated human adipocytes were then assessed for (*B*) ^3^H-2-DOG glucose uptake (N = 6) and (*C*) lactate secretion (N = 6) after 8-Br-cAMP and forskolin treatment for 2 h. For glucose uptake, results shown are normalized mean ± SD of representative experiment. For lactate measurements, results are normalized mean ± SD of three to six biologically independent experiments. In all experiments, N represents biologically independent repeats. One-sample *t* test against one was used for *A*. Ordinary one-way ANOVA statistics was used for *B* and *C*. Holm–Sidak multiple comparison test was used to compare the selected pairs of means. Significance was marked as the pairwise comparisons on the figure. ∗∗∗*p* < 0.001, ∗∗*p* < 0.01, and ∗*p* < 0.05. 8-Br-cAMP, 8-bromine-cAMP; ^3^H-2-DOG, ^3^H-2-deoxy-d-glucose; TXNIP, thioredoxin-interacting protein.

To extend the physiological relevance of these observations, we studied how human primary adipocytes respond to increased cAMP levels. Similar to that we have observed in 3T3-L1 adipocytes, human primary adipocytes degrade TXNIP in response to increased cAMP stimulation (Fig. 7*A*). We also observed increased glucose uptake and lactate production (Fig. 7, *B* and *C*). In conclusion, these results indicate that the cAMP-mediated effects observed in 3T3-L1 adipocytes also are present in primary human adipocytes.

## Discussion

As energy substrates, lipids and carbohydrates are crucial for cellular ATP production. Lipolysis will, in several steps, using a series of lipases, catalyze the breakdown of TAG into FFA and glycerol. FFA will be transported into the mitochondria and undergo successive steps of beta oxidation, which will ultimately generate ATP. Long chain carbohydrates will be broken down to glucose in the small intestine and subsequently taken up by cells *via* the circulation. Glucose will be metabolized to release ATP during glycolysis and in the tricarboxylic acid cycle. Moreover, instead of going directly into the mitochondria for ATP production, pyruvate can be reduced to form lactate, which in turn can be released to the circulation and serve as an important energy source for other cells (25). From a strict point of energy production, both carbohydrates and fats can generate ATP; however, for biosynthetic purposes, carbohydrate carbons are unique in the sense that they are important for the synthesis of biological building blocks, such as purines, *via* the pentose phosphate pathway. This pathway, which makes use of glucose-6-phosphate to produce ribose-5-phosphate, is a precursor for the synthesis of nucleotides. Since mammals lack, or have very low levels of, enzymes required to run the glyoxylate cycle, we cannot, in any significant way, convert fatty acid carbons to carbohydrate carbons. Thus, we depend on carbons from carbohydrates to maintain adequate metabolism and biosynthetic capacity (26).

**Figure 8:**
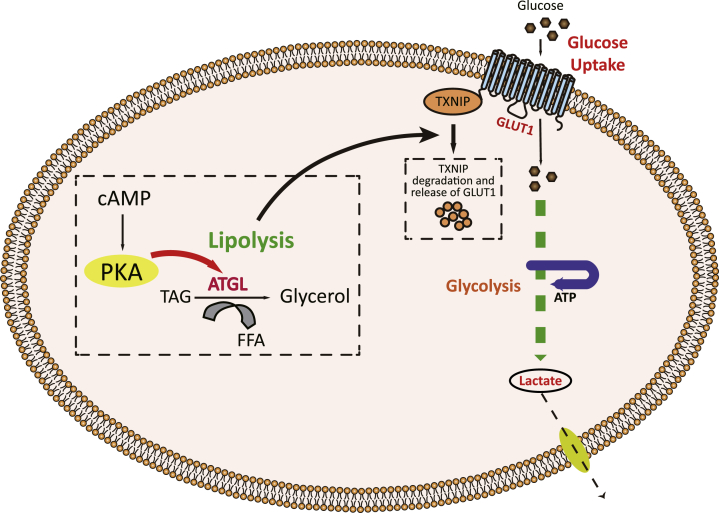
**Schematic representation of cAMP-mediated TXNIP degradation followed by subsequent glucose uptake and lactate secretion.** TXNIP, thioredoxin-interacting protein.

We found that during a situation where cellular cAMP levels are induced and lipases activated, typical of a starvation situation during which insulin levels are very low, there is an enhanced lipase activity with a concomitant increase in glucose uptake (Fig. 1, *D*–*F*). This is supported by a previous report studying glucose uptake in muscle cells in response to adrenergic stimulation (27, 28). Furthermore, we observed, in response to increased cAMP levels, a degradation of TXNIP and the activation of GLUT1, but not GLUT4-mediated glucose uptake. Interestingly, this pathway, which leads to GLUT1-mediated glucose uptake that we show here, depends on the enzymatic activity of ATGL, but not HSL or MGL (Fig. 3*B*). A selective knockdown of ATGL, but not that of HSL or MGL, will decrease TXNIP degradation and GLUT1-mediated glucose uptake (Figs. 3*D* and 4*D*). Moreover, the specific inhibitor of ATGL activity, atglistatin, also decreases TXNIP degradation and GLUT1-mediated glucose uptake (Figs. 2, *A*–*C* and 4*H*). These data support the view that during induced cellular levels of cAMP, and when insulin levels are low, a cAMP-induced and TXNIP degradation–dependent mechanism will ensure the uptake of glucose that is necessary for cellular biosynthetic purposes. To ensure that this mechanism is active when fatty acid is the major energy substrate, the GLUT1-mediated glucose uptake is dependent on ATGL activity (Fig. 8).

Glucose uptake is tightly linked with the expression of TXNIP, which itself is regulated by many different cues, both insulin-dependent and insulin-independent (15, 16). Thus, TXNIP levels influence both the insulin-dependent and insulin-independent arm of glucose uptake. Here, we observed that AMPK and AKT pathways, both of which have been known to regulate glucose uptake and TXNIP degradation, are not linked with this type of TXNIP degradation (Fig 1*I*, *lower panel*). Although many aspects of lipolysis have been studied in detail and are well characterized, recently, new functions of lipolytic products as signaling and transcriptional modulators have been identified (4, 9, 10, 17). In this study, we found that elevated cAMP levels, which are the primary signal for activating cellular lipolysis (19), are associated with degradation of TXNIP—the gatekeeper protein for internalization of GLUTs (15, 16). Mechanistically, we established that ATGL activity is required for TXNIP degradation. TXNIP degradation leads to GLUT1 surface localization and glucose uptake, which is metabolized to lactate. This can be inhibited by either administration of atglistatin or ATGL knockdown. Taken together, the data presented here are compatible with a view that GLUT1 ensures limited glucose uptake needed for biosynthetic purposes in situations where cellular cAMP levels are elevated and GLUT4 is inactive since it functions as an insulin-dependent glucose carrier in the postprandial state. Thus, besides its well-established role in activating lipolysis, cAMP signaling is also critical for GLUT1-mediated glucose uptake.

Our results suggest that adipocytes have an ATGL-dependent mechanism by which glucose uptake can be stimulated. Most of the data have been collected *in vitro* using 3T3-L1 adipocytes. Even though we have replicated the main finding in primary human adipocytes, we do not provide any *in vivo* data. Another limitation of our study is that apart from 3T3-L1 adipocytes and human primary adipocytes, we only test two more cell types C2C12 and 3T3-L1 preadipocytes. Hence, it is possible that other cell types not tested here also use this ATGL-dependent pathway to regulate their glucose uptake.

In conclusion, we would like to suggest that in adipocytes, GLUT1 ensures limited glucose uptake during starvation, which is typically accompanied with increased cAMP levels and activated lipolysis, whereas GLUT4 functions in a postprandial milieu to stimulate the uptake of ingested glucose.

## Experimental procedures

### Materials

Puromycin, dexamethasone, isobutyl methylxanthine, rosiglitazone, bovine insulin, 8-bromoadenosine 3′,5′-cyclic monophosphate sodium salt (8-Br-cAMP) (B7880), atglistatin (SML1075), dorsomorphin, polybrene (hexadimethrine bromide), 2-deoxyglucose, SB 216763 (GSK inhibitor) (S3442-5MG), and H89 (B-1427) were purchased from Sigma–Aldrich. Antibodies against TXNIP (#14715), ATGL (#2138) Akt, phospho-Akt, phospho-AS160 (Thr642), and actin-horseradish peroxidase (#12262) were obtained from Cell Signaling Technologies. GLUT1 and GLUT4 antibody Akt inhibitor MK-2206 was purchased from Cayman. Super Signal West Pico and Super Signal West Dura chemiluminescent substrate were obtained from Pierce (Thermo Fisher Scientific). RNeasy mini kit was purchased from Qiagen. Radiochemicals and ^3^H-DOG were purchased from from Perkin Elmer.

### Cell culture and generation of stable cells

3T3-L1 fibroblasts (American Type Culture Collection) were maintained in culture and differentiated into adipocytes as previously described. Lipofectamin 2000 and RNAiMAX (Life Technology) were used for transfections of mammalian cells according to the manufacturer's protocol. Retroviral particles were produced in human embryonic kidney 293T cells after transfection with a standard combination of expression and packaging vectors (29). 3T3-L1 fibroblasts (30–40% confluent) were infected with respective virus particles for 48 h. After infection, stably expressing cells were selected in puromycin-containing culture media.

Primary human preadipocytes were purchased from Promo cell (C-12730) and cultured according to manufacturer's instructions. Primary human preadipocytes were differentiated using differentiation media (811D-250) according to their instruction. Experiments were performed after full differentiation protocol and 95% adipocyte differentiation.

### Plasmids, siRNAs, and shRNAs

Full-length human ATGL (MR226260) and TXNIP (MR206196) with C-terminal triple FLAG tag (3XFLAG) in pcDNA3.1 vectors were purchased from Origene and subcloned into retroviral pBabe-puro (ATGL) or pRetroXTRE3G (TXNIP) vectors for OE. FLAG-rGLUT1 vector was ordered from Addgene (#89571) and HA-GLUT4-GFP construct was kindly gifted from Prof. McGraw Lab, Weill Cornell Medicine, NY, USA. Both GLUT1 and GLUT4, along with their tagged versions (FLAG and HA), were subcloned into pBabe-puro vector for stable cell generation. ATGL (SASI_Mm01_00033377), HSL (SASI_Mm01_00090382), MGL (SASI_Mm01_00106711), TXNIP siRNA (SASI_Mm01_00138870), GLUT1 (SASI_Mm01_00068163), and GLUT4 (SASI_Mm01_00032134) together with scramble siRNA (SIC001) as a control were purchased from Sigma.

pLKO.1 vector was purchased from Addgene (#10878), and the following set of shRNAs were cloned using standard procedures. After confirming shRNA clone presence, constructs were used for virus particle production, and subsequent stable cell generation was performed as mentioned in the previous section. Empty vector was used as a control throughout the experiments.

### shRNA sequence for mouse ATGL

Forward: CCGGCATCTCCCTGACTCGTGTTTCCTCGAGGAAACACGAGTCAGGGAGATGTTTTTG

Reverse: AATTCAAAAACATCTCCCTGACTCGTGTTTCCTCGAGGAAACACGAGTCAGGGAGATG

### shRNA sequence for mouse GLUT1

Forward: CCGGTGAGGAGTTCTACAATCAAACCCTCGAGGGTTTGATTGTAGAACTCCTCATTTTTG

Reverse: AATTCAAAAATGAGGAGTTCTACAATCAAACCCTCGAGGGTTTGATTGTAGAACTCCTCA

### Glucose uptake assay

Glucose uptake in differentiated 3T3-L1 adipocytes was assessed by analysis of 2-[1,2-^3^H(N)]-DOG taken up by cells, essentially as described earlier (29). Assays were performed in Krebs–Ringer phosphate buffer supplemented with 0.2% bovine serum albumin (downstream media). Briefly, cells were washed in downstream media and incubated in downstream media containing 5 mM glucose for 2 h. After completion of 2 h, cells were washed 2 times with downstream media without glucose, followed by quick addition of DOG [1,2-^3^H(N)] (0.125 μCi/well) and 50 μM cold DOG and incubated for 10 min. Cells were then immediately washed 3 times with chilled PBS and lysed in 0.1% Triton-100. A small aliquot from the lysate was taken for protein measurement, and the remainder transferred to scintillation fluid for radioactive counting. The CPM values were normalized to the total cellular protein level for each well.

### GLUT1 and GLUT4 surface localization assay

Differentiated adipocytes stably expressing tagged GLUT1 or GLUT4 were serum starved for 2 h in Dulbecco's modified Eagle's medium serum-free media. During this starvation, 8-Br-cAMP was incubated throughout starvation to assess GLUT1/GLUT4 surface localization. As a positive control for GLUT4, insulin was added during the last 20 min to trigger GLUT4 translocation. To see the effect of atglistatin on GLUT1 surface levels, cells were preincubated with atglistatin before adding 8-Br-cAMP. After completion of serum starvation, cells were immediately washed with chilled PBS and fixed with 3.7% formaldehyde for 7 min. Cells were washed 3 times with PBS and incubated with FLAG or HA antibody for 1 h at 37 °C. Cells were washed 3 times with PBS and incubated with secondary horseradish peroxidase–linked antibody for 1 h. Cells were washed 2 to 3 times, and 3,3′,5,5′-tetramethylbenzidine substrate was added. After 10 to 20 min of development, the reaction was stopped with 0.1 N HCl, and absorbance was measured at 450 nm. Total FLAG/HA (total GLUT1/GLUT4) was determined using permeabilization with Triton X-100 (0.01%) and subtracted from surface values to obtain surface-to-total ratio. Control cells were taken as a blank control and subtracted from all values.

### RNA and protein analysis

For Western blots, cells were lysed in CST buffer (Cell Signaling Technology, #9803), supplemented with protease and phosphatase inhibitor cocktails according to the manufacturer's recommendations. Protein concentrations were measured using the BCA protein assay kit (ThermoFisher). Proteins were separated by SDS-PAGE on NuPAGE Novex 4 to 12% Bis–Tris protein gels and transferred to polyvinylidene fluoride membranes. Specific proteins were detected with the indicated primary antibodies. Horseradish peroxidase–coupled secondary antibody was visualized with SuperSignal West Pico or SuperSignal West Dura Chemiluminescent Substrates on a LAS-4000 Luminescent Image analyzer (FujiFilm). Density of the bands was quantified using ImageJ software (National Institutes of Health), and results were expressed as fold changes compared with the signal observed in the control cells (infected with empty vector) after normalization to actin level.

Total RNA from adipocytes was isolated using RNeasy mini kit according to the manufacturer's instructions. Reverse transcription of 1 μg of total RNA was carried out using the First-Strand cDNA synthesis kit. Expression levels of specific mRNAs were quantified by real-time PCR using Power SYBR green PCR Master Mix on ABI Prism 7900 Sequence Detection System (Applied Biosystems, Thermo Fisher Scientific) and normalized to 36B4. All samples were analyzed in triplicate, and mean values were calculated.

### Lactate, ATP, and glycerol measurements

Lactate was measured in cAMP-treated supernatant samples using EnzyChrom L-lactate Assay Kit (ECL-1000) as per manufacturer's protocol. Total cellular ATP content was determined using EnzyLight ATP Assay Kit (EATP-100) according to manufacturer's protocol. Glycerol measurements were performed with supernatant samples taken after various treatments and secreted glycerol measured using the Glycerol Assay Kit (MAK117) according to manufacturer's protocol.

### Statistical analyses

Data are expressed as mean ± SD. *p*-Values were calculated by ANOVA and Student's *t* test. *p*-Values of <0.05 were defined as statistically significant.

## Data availability

All the data are contained within the article.

## Conflict of interest

The authors declare that they have no conflicts of interest with the contents of this article.
