# Supplementary material for: ATGL activity regulates GLUT1-mediated glucose uptake and lactate production via TXNIP stability in adipocytes
Source: J Biol Chem. 2021 Jan 27;296:100332. doi: 10.1016/j.jbc.2021.100332 (PMC7949114; doi:10.1016/j.jbc.2021.100332)
Supplement: Supplemental Figures S1–S10 [file mmc1.pdf]

SUPPORTING INFORMATION

ATGL activity regulates GLUT1-mediated glucose uptake and lactate production via TXNIP stability in adipocytes

Muheeb Beg, Wei Zhang, Andrew C. McCourt and Sven Enerbäck

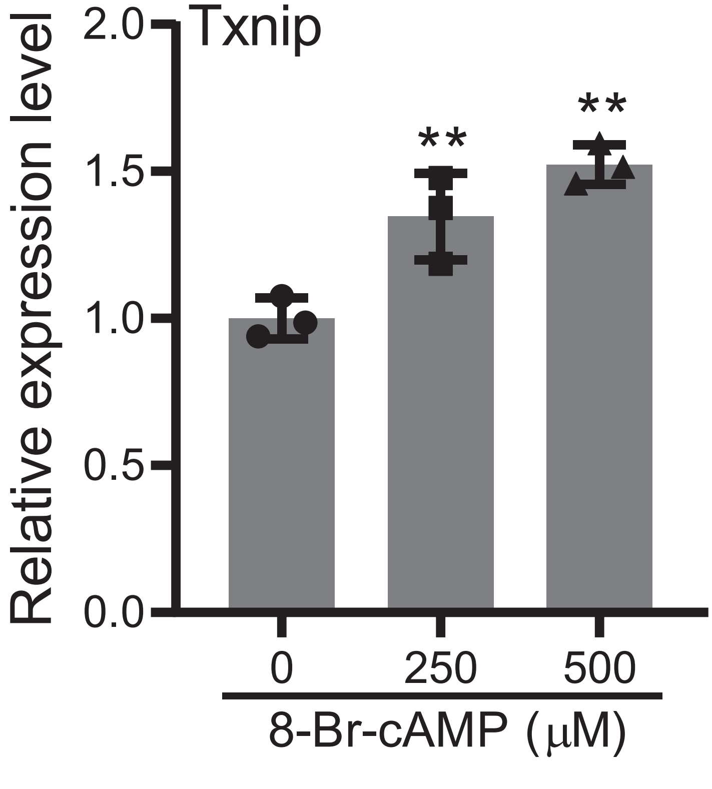

Supp. Fig 1: Differentiated adipocyte cells were treated with various concentration of 8-Br-cAMP for 2h followed by measurement of TXNIP relative mRNA using qPCR. N=3. Ordinary one-way ANOVA statistics was used. Holm-Sidak multiple comparison test was used to compare the selected pairs of means. Significance was marked as the pairwise comparisons on the figure. ***P < 0.001, **P < 0.01, *P < 0.05.

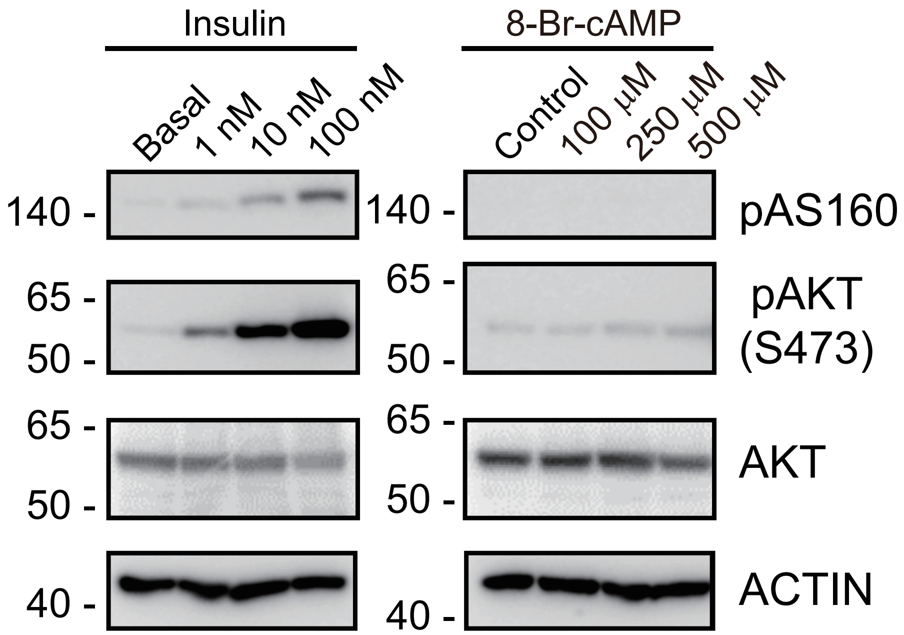

Supp. Fig 2: Differentiated adipocyte cells were subjected to immunoblots for the insulin-signaling pathway after stimulation with dose dependent insulin (20 min) or cAMP (2h). N=1

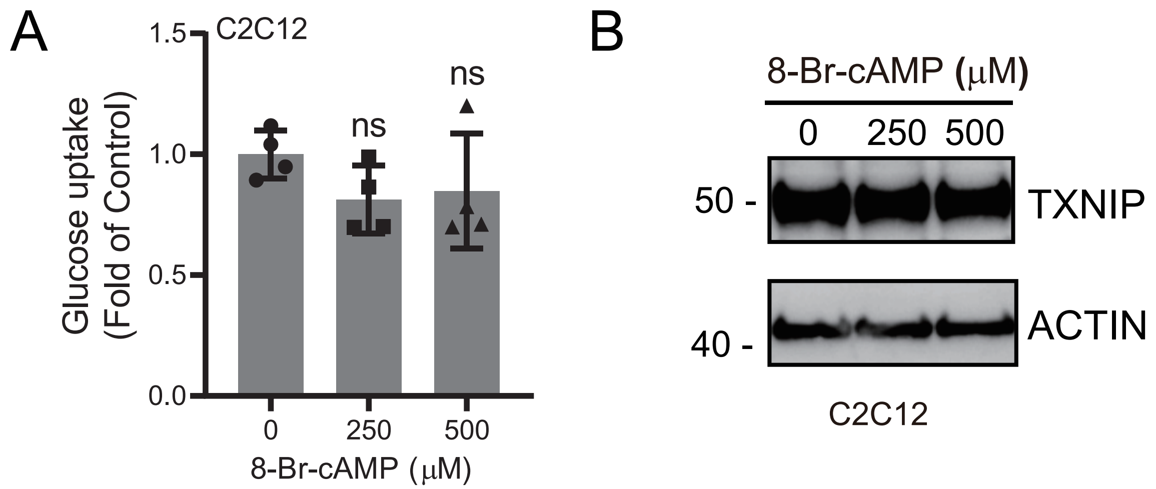

Supp. Fig 3: C2C12 myoblast were stimulated with 8Br-cAMP for 2h and cells were subject to glucose uptake measurement **(A)** and TXNIP immunoblot expression **(B)**. N=2. Ordinary one-way ANOVA statistics was used. Holm-Sidak multiple comparison test was used to compare the selected pairs of means. Significance was marked as the pairwise comparisons on the figure. ***P < 0.001, **P < 0.01, *P < 0.05, ns - no significance.

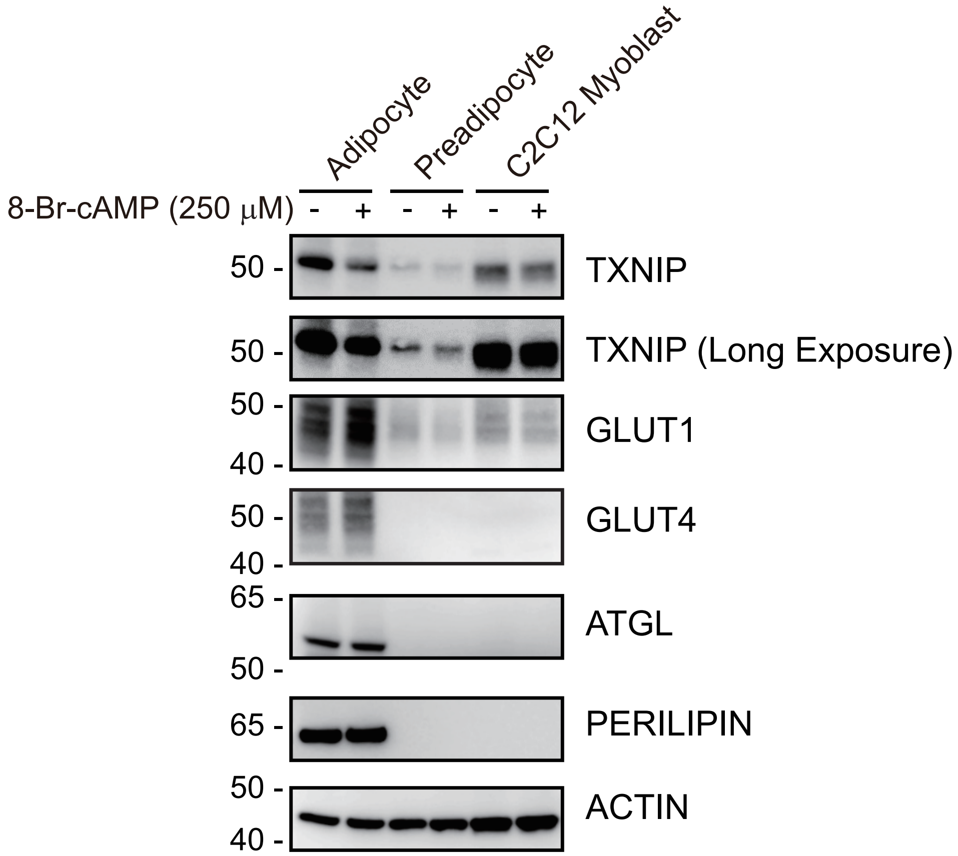

Supp. Fig 4: Differentiated adipocyte, preadipocyte and C2C12 cells were stimulated with 8-Br-cAMP followed by immunoblot expression analysis of TXNIP, GLUT1, GLUT4, ATGL and perilipin. Representative blots were show from 3 biological independent repeat experiment.

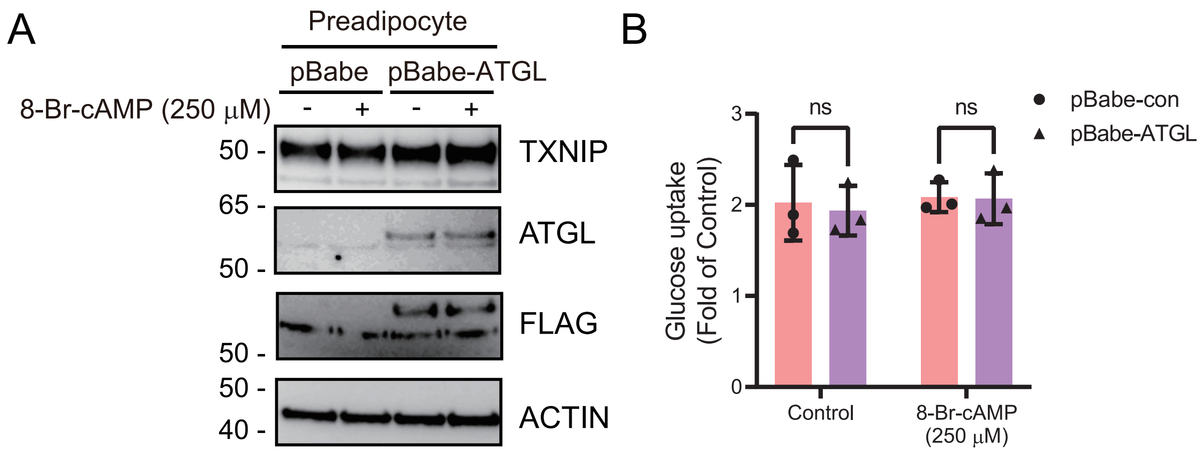

Supp. Fig 5: Preadipocytes stably expressing FLAG-ATGL or empty pBabe vector were stimulated with 8-Br-cAMP followed by **(A)** immunoblot analysis and **(B)** glucose uptake measurement. N=2. Two-way ANOVA statistics was used. Holm-Sidak multiple comparison test was used to compare the selected pairs of means. Significance was marked as the pairwise comparisons on the figure. ***P < 0.001, **P < 0.01, *P < 0.05, ns - no significance.

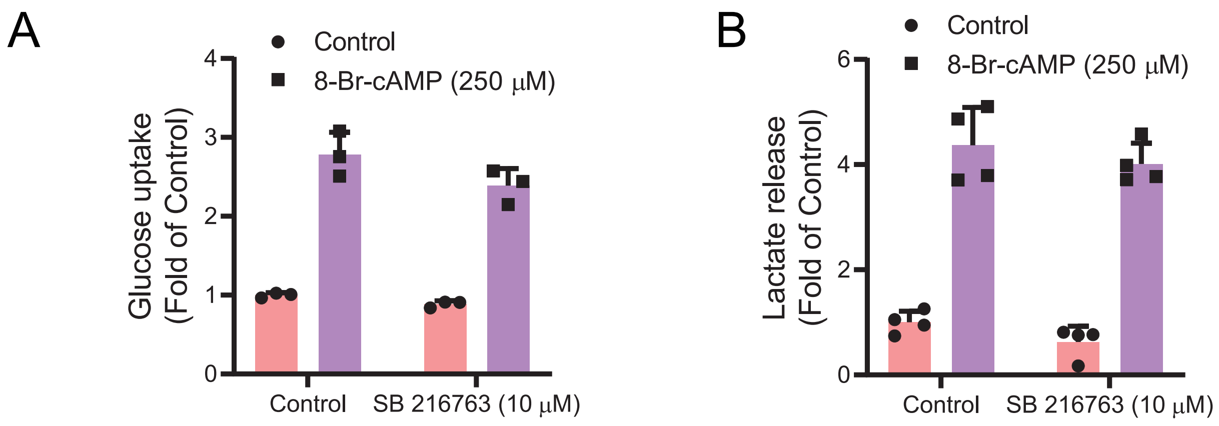

Supp. Fig 6: Differentiated cells were pre-treated with GSK3 inhibitor (SB216763) followed by co-incubated with cAMP for 2h and then **(A)** glucose uptake or **(B)** lactate secretion analyses were performed**,** N=2.

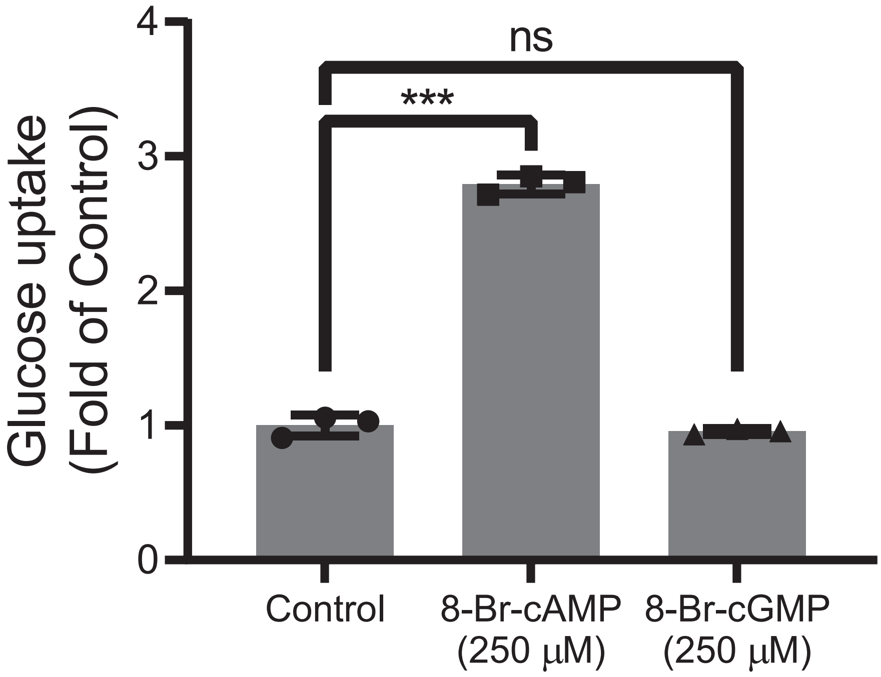

Supp. Fig 7: Differentiated cells were stimulated with 8Br-cAMP or 8-Br-cGMP for 2h followed by glucose uptake determination, N=2. Ordinary one-way ANOVA statistics was used. Holm-Sidak multiple comparison test was used to compare the selected pairs of means. Significance was marked as the pairwise comparisons on the figure. ***$P < 0.001$, **$P < 0.01$, *$P < 0.05$, ns - no significance.

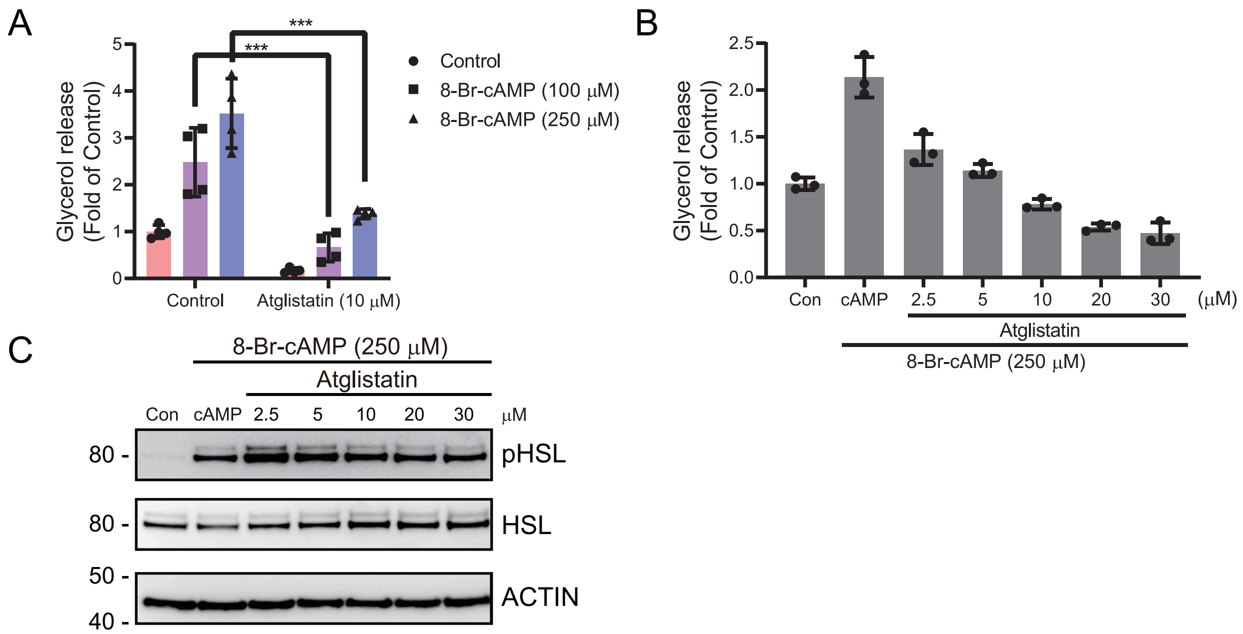

Supp. Fig 8: Differentiated cells were pretreated with atglistatin (10μM) for 20 min followed by 2h co-incubation with **(A)** different concentration of 8-Br-cAMP followed by measurement of glycerol in supernatants (N=3). Similarly, **(B)** glycerol measurement was performed using different concentration of atglistatin in presence of cAMP (250μM) (N=2). **(C)** In similar setting protein lysates were taken to assess the effect of Atglistatin on cAMP mediated HSL phosphorylation (Ser563) (N=2). Two-way ANOVA statistics was used. Holm-Sidak multiple comparison test was used to compare the selected pairs of means. Significance was marked as the pairwise comparisons on the figure. ***P < 0.001, **P < 0.01, *P < 0.05.

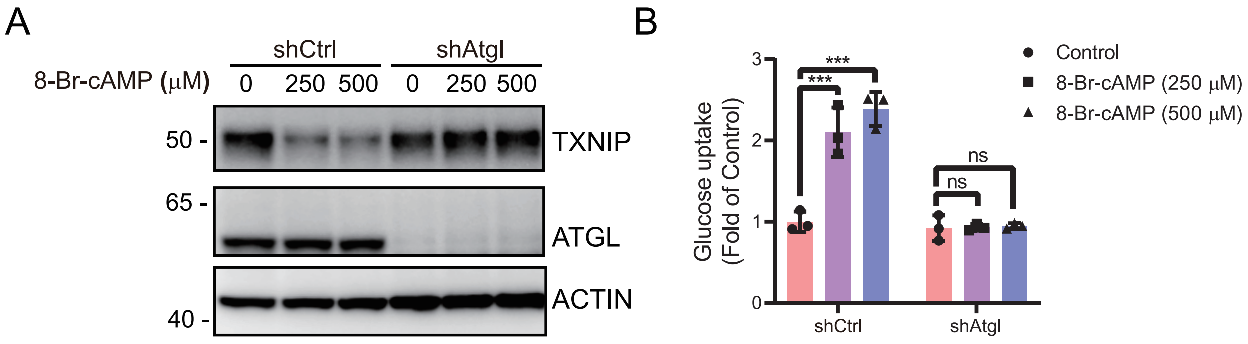

Supp. Fig 9: Differentiated cells stably expressing shRNA of ATGL were treated with 8-Br-cAMP for 2h followed by **(A)** immunoblot analysis TXNIP and ATGL and **(B)** measurement of glucose uptake**.** Representative blots and glucose uptake experiment shown out 4-5 biological independent experiments. Two-way ANOVA statistics was used for glucose uptake. Holm-Sidak multiple comparison test was used to compare the selected pairs of means. Significance was marked as the pairwise comparisons on the figure. ***$P < 0.001$, **$P < 0.01$, *$P < 0.05$, ns - no significance.

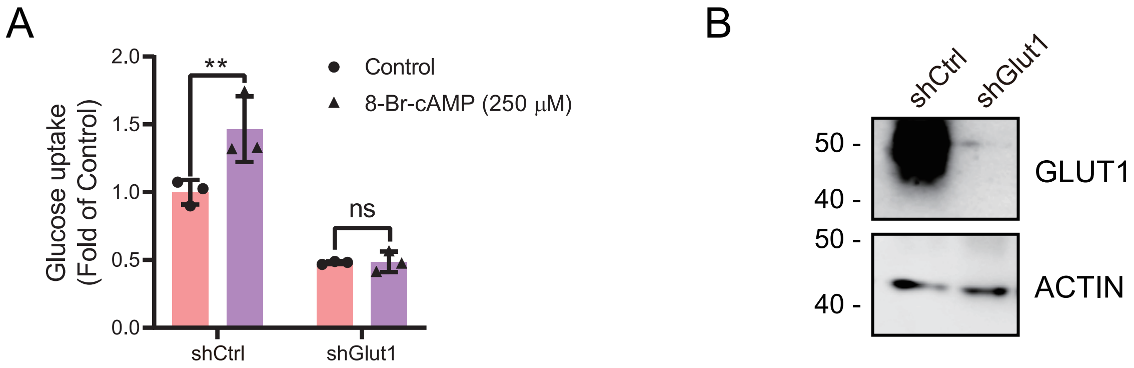

Supp. Fig 10: (A) Differentiated cells stably expressing shRNA of GLUT1 were treated with 8-Br-cAMP for 2h followed by measurement of glucose uptake (N=4). **(B)** Knockdown of GLUT1 was confirmed using immunoblot analysis. Representative blots and glucose uptake experiment shown out 4-5 biological independent experiments. Two-way ANOVA statistics was used for glucose uptake. Holm-Sidak multiple comparison test was used to compare the selected pairs of means. Significance was marked as the pairwise comparisons on the figure. ***$P < 0.001$, **$P < 0.01$, *$P < 0.05$, ns - no significance.
